# Facilitators and Barriers to the Implementation of Family Integrated Care in Ontario Level II Neonatal Intensive Care Units

**DOI:** 10.3390/children12111548

**Published:** 2025-11-16

**Authors:** Ayah Al Bizri, Mariana Bueno, Vibhuti Shah, Fabiana Bacchini, Douglas M. Campbell, Karen M. Benzies, Karel O’Brien

**Affiliations:** 1 Department of Paediatrics, Mount Sinai Hospital, 600 University Avenue, Toronto, ON M5G 1X5, Canada; ayah.albizri@sinaihealth.ca (A.A.B.); vibhuti.shah@sinaihealth.ca (V.S.); 2 Lawrence Bloomberg Faculty of Nursing, University of Toronto, 155 College Street, Suite 130, Toronto, ON M5T 1P8, Canada; mariana.bueno@utoronto.ca; 3 Canadian Premature Babies’ Foundation, Toronto, ON M8X 1Y3, Canada; fabiana@cpbf-fbpc.org; 4 Neonatal Intensive Care Unit, St Michael’s Hospital, Unity Health Toronto, Toronto, ON M5B 1W8, Canada; douglas.campbell@unityhealth.to; 5 Department of Pediatrics, University of Toronto, Toronto, ON M5T 1P8, Canada; 6 Li Ka Shing Knowledge Institute, Unity Health Toronto, Toronto, ON M5B 1W8, Canada; 7 Allan Waters Family Simulation Program, St Michael’s Hospital, Unity Health Toronto, Toronto, ON M5B 1W8, Canada; 8 Faculty of Nursing, Departments of Pediatrics and Community Health Sciences, Cumming School of Medicine, University of Calgary, 2500 University Drive NW, Calgary, AB T2N 1N4, Canada; benzies@ucalgary.ca

**Keywords:** FICare, Level II NICU, Preterm Birth, CFIR

## Abstract

**Highlights:**

**What are the main findings?**

• CFIR was a useful framework for the identification of facilitators and barriers to implementing FICare in Ontario’s Level II NICUs.
• There is variability in the readiness of Level II NICUs to implement FICare based on NICU preparedness, capacity, population served, and geographical distribution.

**What is the implication of the main finding?**

• The knowledge gained using the CFIR framework will guide the implementation of FICare in Ontario.
• Implementation of FICare will require specific attention to the context of NICUs in the province, such as the population served and the geographical situation.

**Abstract:**

**Background/Objectives**: In Ontario, approximately 8% (11,000) of infants are born preterm (22–<37 weeks gestation) each year. Many of these infants are cared for in a Level II Neonatal Intensive Care Unit (NICU). Family Integrated Care (FICare), an innovative model of care, aims to facilitate the involvement of parents in the care of their infants in NICUs. The aim of this study was to gain a better understanding of the general and specific needs of Level II NICUs in Ontario prior to implementation of FICare. **Methods**: Using a cross-sectional study design, two surveys (leadership and site resources) were developed using the Consolidated Framework for Implementation Science Research’s innovation, inner setting, and outer setting constructs and distributed to Level II NICUs medical and nursing leaders. **Results**: The surveys were sent to 44 Level II NICUs in Ontario, of which 24 hospitals (55%) responded. Key facilitators to implementation of FICare in Level II hospitals in Ontario were leadership interest, availability of staff and parent volunteers, and existing policies to support implementation. The identified barriers were lack of financial resources for new initiatives, skepticism in FICare’s ability to save costs, need for tailored implementation due to variability in NICU characteristics, and the lack of environmental support for prolonged parental presence. **Conclusions**: This study has confirmed the interest of many Ontario level II NICUs in implementing FICare and variability in their readiness for implementation based on the identified facilitators and barriers.

## 1. Introduction

Approximately 11,000 infants are born preterm (22–<37 weeks gestation) each year in Ontario, Canada [1]. Of those, about 80% are born between 33 and 36 weeks gestational age, with many of these infants receiving care in a Level II NICU. Level II NICUs provide care to a variety of preterm infants from 30–34 weeks gestational age and/or birth weight of >1000–1500 g as well as term infants who require intensive care monitoring [2,3]. These newborns are cared for under the Ontario Health Insurance Plan, with no extra cost to Ontario residents [4]. Family Integrated Care (FICare), an innovative model of care, aims to facilitate the involvement of parents in the care of their infants in neonatal intensive care units (NICUs) [5,6,7]. This is particularly important to Ontario-resident parents who are entitled to 63 weeks of job-protected parental leave and have the opportunity to stay by their infant’s side in the NICU [8]. Based on the principles of family-centered care, the model was developed to support parents in becoming ’primary caregivers’ for their infants in the NICU through the mentorship and support of the clinical team [5,6,7]. This model seeks to address the stressful and traumatic impacts of the NICU environment on infants and their families that can lead to a higher risk of maternal depression, anxiety, parent-infant separation, infant complications and developmental delays, and stress [9,10,11,12,13].

The effectiveness of FICare in Level III NICUs has been well demonstrated in two cluster randomized controlled trials (cRCTs) [9,10]. In the first cRCT involving 26 tertiary care NICUs in Canada, Australia and New Zealand, results showed improved neonatal outcomes compared to usual care, such as improved daily weight gain (26.7 g vs. 24.8 g), higher rates of high-frequency (6 feeds a day) breastfeeding at discharge (70% vs. 30%), and decreased parental stress and anxiety [14]. The second cRCT conducted in 11 NICUs in China showed shorter duration of hospital stay (28 vs. 35 days), reduced duration of oxygen supplementation (13.1 vs. 21.4 days), lower rates of nosocomial infections (4.13 vs. 5.84/1000 hospital days), and increased breastfeeding rates (87.3% vs. 55.8%) when compared to standard care [15]. Additionally, the outcomes of FICare were noted to persist beyond discharge from the hospital, by reducing maternal stress and improving child self-regulation at 18 months [16,17]. Recognizing the positive impact on both neonatal and parental outcomes, FICare has been widely adopted in clinical practice in Level III NICUs [18,19].

FICare implementation in Level II NICU has the potential to support the majority of preterm births and have a greater effect on infants, families, and the health system. The outcomes associated with FICare implementation in Level II NICUs in Canada have been promising [20]. In a cRCT from Alberta, adaptation of FICare model was associated with improved infant health by decreasing length of stay by 2.5 days without increasing rates of readmissions or emergency department visits compared to standard care [20]. Despite the surveillance and extensive support (site visits, liaisons with super users, fidelity audits) provided by the research team, the uptake varied by site, where one site struggled due to challenges with community pediatrician coverage for structured bedside rounds, a population with high social risk, and high staff turnover [20]. This led investigators to undertake a process evaluation to understand the key influences on the implementation [21]. The evaluation identified a receptive implementation climate, compatibility of the intervention with individual and organizational practices, availability of resources and access to knowledge and information, key stakeholders’ engagement, engagement of intervention participants, and reflecting and evaluating on implementation progress and patient and family outcomes as key facilitators of FICare implementation [21]. It also identified the intervention design quality and packaging, relative priority of the intervention in relation to other initiatives, and the learning climate within the organization as key barriers to FICare implementation. Accordingly, the investigators highlighted the need for site-specific consultations to mitigate the barriers, emphasizing the importance of adapting care models to local contexts [21].

The different local context of Level II NICUs in Ontario poses challenges to the implementation of FICare as compared to Alberta. For example, there are 44 Level II NICUs in Ontario, serving diverse populations with varied geographic distributions (urban and rural), nurse-to-patient ratios, and physician staffing patterns [2]. Accordingly, there was a need to identify and understand the Ontario-specific facilitators and barriers to implementing FICare in Level II NICUs prior to proceeding with a wide-scale implementation study. Therefore, the aim of this study was to gain a better understanding of the general and specific needs of each individual Level II NICU in Ontario prior to the implementation of FICare.

## 2. Materials and Methods

A cross-sectional study design was used to understand the potential facilitators and barriers to implementing FICare in Level II NICUs in Ontario. Our population of interest was the province’s 44 Level II NICUs identified by the Ministry of Health of Ontario. Specifically, we sought the feedback of NICU medical and nursing leaders who could provide an overview of their NICUs readiness through the Ontario FICare (ON-FICare) Level II NICU surveys.

### 2.1. Measurement Tools

The ON-FICare Level II NICU surveys were developed by this study’s steering committee based on the Consolidated Framework for Implementation Research (CFIR) (2009) [22]. Our population of interest is the individual NICU. Due to the aims of this study, we focused on three of CFIR’s constructs: intervention, inner setting, and outer setting, to guide the survey development [22]. Accordingly, the individual and process constructs were not used or reported in this study. Members of the steering committee consisted of FICare researchers (KO, VS), an implementation scientist (MB), an investigator from Alberta FICare (KB), an investigator from a Level II NICU (DC), a knowledge user (Provincial Council for Maternal and Child Health), and a parent volunteer (FB). Surveys were developed and reviewed by the members of the steering committee to identify potential facilitators and barriers to implementing FICare in Level II NICUs. We developed two separate surveys guided by the question topics and flow, and to facilitate the usability of the surveys as they might be filled out by different members of the unit’s leadership, medical, and nursing teams. The leadership survey, comprising 32 questions, aimed to understand a unit’s general attitudes, procedures, environment, and available support for implementing FICare from a leadership perspective. The site resource survey, comprising 66 questions, collected information on NICU demographics, staffing, and available facilities for parents to understand the unit’s current situation and practices, as well as what interventions might be required to implement FICare. The surveys were created on a specific platform, created for the Canadian Neonatal Network, and hosted on their website. The surveys were pilot-tested for usability and clarity by one of the Level II NICUs in Ontario. No changes were implemented to the leadership survey, while the site resource survey was modified for clarity by changing the formatting of some questions (See Supplementary Material for the leadership and site resource surveys). This study was approved by the Research Ethics Board, Mount Sinai Hospital (REB number: 23-0103-E, approved on 24 October 2023) and reported using the STROBE statement.

### 2.2. Study Procedure

Surveys were distributed to the medical and nursing leaders in each of the 44 Level II NICUs in November 2023. Three weekly reminders were sent to hospitals that did not complete the surveys within the first month. This was followed by email reminders every 2–3 weeks until the end of January for a total of seven reminders. Additionally, Chiefs of Paediatrics from units that did not respond to the surveys by phone or personal email were contacted to introduce FICare and the importance of completing the surveys. Hospitals were also contacted and asked to complete the missing survey when only one survey had been completed, and to fill in missing information when survey questions were unanswered. At the initiation of the study, we planned to share the results with the participating NICUs through a dissemination webinar to validate the findings and gauge interest in FICare implementation.

### 2.3. Statistical Analysis

Data were downloaded from the Canadian Neonatal Network website, and extraneous non-data fields were removed. Data was then examined for missing values and patterns of missing values. Microsoft Excel 2013 was used to manage data and calculate descriptive statistics. Frequencies and percentages were used to describe the characteristics of NICUs (number of beds, number of nursing staff, staffing model, and urban/rural location), as well as to report survey data.

## 3. Results

Respondents from 24 hospitals (55%) completed the surveys. Of those, 19 (79%) completed both the leadership and site resource surveys, while five completed the leadership survey only. Results are organized by the three CFIR constructs captured on the survey. To showcase all the results received from Level II NICUs, all responses are presented, and missing data is indicated when applicable.

### 3.1. Inner Setting

#### 3.1.1. Structural Characteristics

Table 1 presents an overview of the characteristics of the 19 Level II NICUs that completed the site resource survey. Around half of the NICUs were 10–20 years old and had an open-concept design. The majority of NICUs had fewer than 20 beds and 500 admissions, with an average occupancy rate ranging between 50% to 80%. In 2022, the number of patient days averaged 3703, and the average length of stay was 8.9 days. Of the respondents who reported NICU resources, 84% reported having facilities for families, defined as having a parent lounge, comfortable seating space near the bedside, a kitchen, or a dedicated sleep/hostel room. However, the leadership survey identified the inadequacies of their environment to support FICare implementation as a barrier, where only 54.2% reported having adequate space, 58.3% reported an environment encouraging parent presence, and 41.7% reported having a space for families to socialize or attend educational sessions (Table 2).

#### 3.1.2. Implementation Climate

Almost all hospitals (95.8%) reported a unit work climate supportive of family-centered care practices, leadership supporting units in implementing practice changes to support families, and policies that encourage parents’ presence with their babies. When asked about specific hospital policies, a third of hospitals reported unit procedures (NICU admission and daily patient bedside rounds) that disturb continuous parent engagement and participation by asking parents to temporarily leave the NICU.

Table 3 reports current parents’ participation practices in Level II NICUS, where all hospitals (100%) encourage skin-to-skin contact, engaging parents in infants’ care activities, and parents participating in decision-making/discharge planning of their newborn. Around 89% encourage parents to be present for rounds and 78% allow parents to develop care plans with their bedside nurse.

#### 3.1.3. Readiness for Implementation

1. Leadership engagement: Results from the leadership survey indicated that 91.7% of hospitals were interested in implementing the FICare model, and 75% reported unit and institutional interest and support for implementing and maintaining FICare (Table 2).
2. Available resources: Although 75% of hospitals reported lacking the financial resources to support new initiatives, the majority reported the ability to support champions to facilitate uptake (80%) and staff education (75%) necessary to sustain FICare (Table 2). Around two thirds of hospitals reported having an adequate staff-to-patient ratio to support FICare (62.5%). Reported staff characteristics that could arise as concerns in FICare implementation include the percentage of new graduate nurses (58.3%), part-time working nurses (29%), and rapid staff turnover (29%) (Table 4). Regarding parental support, 72% of NICUs reported having a dedicated social worker, while 28% had a social worker available by consultation. Moreover, 26.3% and 21.1% of hospitals reported having a veteran parent (parent buddy) and parent-to-parent support, respectively, in their units (Table 5).

### 3.2. Outer Setting

#### Patient Needs and Resources

The hospitals reported serving a variety of inner city, suburban, rural, and new immigrant populations. Hospitals identified characteristics of families that need to be highlighted in the implementation of FICare at their site (Table 4). Families with other children and lack of extended family or social support were identified by 91.7% of hospitals as important population to tailor FICare implementation for, followed by Families who do not speak English as their first language (70.8%), Families with housing insecurity (66.7%) and single mothers (66.7%).

### 3.3. Intervention Characteristics

#### 3.3.1. Evidence Strength and Quality

More than half of the NICUs reported positive perceptions of FICare’s quality and validity. Specifically, 75% of units reported that medical staff support FICare, 67% of units reported that their nursing staff support FICare, while 50% of NICUs reported having allied health staff that support FICare. Moreover, 88% of units reported that their staff are motivated to integrate parents into the multidisciplinary team.

#### 3.3.2. Relative Advantage

When asked about ON-FICare’s potential for creating cost savings for the Women and Children’s program, only 41.7% agreed, 45.8% were undecided, while 12.5% disagreed that family-centered care programs can decrease costs (Table 2).

#### 3.3.3. Complexity

Acknowledging that ON-FICare is a complex intervention, we asked respondents to identify difficulties they anticipate in the implementation of ON-FICare (Table 6). More than half of the units reported that supporting parent presence would be difficult to implement. Alternatively, almost all (95.8%) NICUs reported ease of coaching parents, and the majority of units reported ease in supporting parent participation (79.2%) and implementing standardized education (70.8%).

### 3.4. Dissemination Webinar

The survey results were shared with the sites through a dissemination webinar, and the sites confirmed that the findings were consistent with their current practices and their interest in implementing ON-FICare.

## 4. Discussion

In this cross-sectional study, of the 44 Level II NICUs in Ontario, respondents from 19 sites completed a site resource survey, and 24 completed a leadership survey. The surveys identified several barriers and facilitators to FICare implementations in Level II NICUs in Ontario.

### 4.1. Inner Setting

Several potential facilitators for FICare implementation in Level II NICUs were identified under the inner setting domain. The units that responded to the survey expressed interest in implementing FICare and reported having the ability to support necessary staff education and champions to achieve this goal. Most of the participating units also indicated that they already have policies in place to support FICare implementation, such as encouraging family presence in NICU and family involvement in infant care activities, confirming the compatibility of FICare with their individual and organizational practices. Additionally, many of the hospitals reported having available staff and parent volunteer resources for FICare implementation, including adequate staff-to-patient ratio, social work support, and, to a lesser extent, parent volunteer support. These three facilitators were identified as key for successful implementation by Benzies et al. in their study of Alberta FICare [21].

This study also examined the many contextual factors of the inner setting that are known from the Alberta FICare study to have mixed influence on implementation and would need to be considered in the implementation plan [21]. These factors include a lack of financial resources for new initiatives, which was reported by many NICUs. Although the implementation of FICare is supported by the Provincial Council for Maternal and Child Health and their standards for newborn care, there is currently no identified financial support from the provincial health body for implementation. Additionally, Ontario’s large number and geographically dispersed Level II NICUs vary by age, bed size, and occupancy rates, necessitating that the implementation is tailored to the unit characteristics. This is further highlighted by the varied support provided for families who live at a distance from the units and the lack of environmental support for prolonged parental presence reported by half of the units. This barrier, the out-of-pocket cost of living a long distance from the NICU, was also identified by Alberta FIcare as a financial stressor [23]. In addition, a third of the hospitals reported that their current routine NICU processes, particularly around bedside rounds, might make it difficult for parents to participate in rounds, and this issue would need to be addressed in some way.

### 4.2. Outer Setting

In addition to the barrier introduced by Ontario’s large number and widely geographically dispersed Level II NICUs, as explained above, the variability in the populations these NICUs serve further confirms the need for a tailored implementation of ON-FICare. Moreover, the lack of resources to acknowledge the population’s diversity and needs, especially for groups with higher social risks, can hinder intervention uptake as was observed in Alberta FICare [20].

### 4.3. Intervention Characteristics

Several facilitators were identified under the intervention characteristic domains. The first facilitator is the positive perceptions of FICare’s quality and validity as observed from medical, nursing, and allied health staff supporting FICare implementation and integration of parents into the multidisciplinary team. Additionally, despite the complexity of the intervention, most units consider it would be easy to coach parents and support their participation in care, as they currently have several policies encouraging parent participation, such as skin-to-skin contact, baby care activities, and involvement in decision-making and discharge planning. However, the lack of financial resources to support the implementation of FICare was perceived as a barrier. More than half of the units were skeptical about FICare’s ability to reduce costs, despite evidence from the Alberta FICare study [18].

Using the CFIR framework at the pre-implementation stage of FICare in Level II NICUs in Ontario enabled the identification of factors that may influence the success or failure of the implementation. This exploration will enable the development of an intervention that not only aligns with the constructs of CFIR’s innovation domain, but also considers determinants related to the inner and outer setting domains. Proactively addressing these factors will also potentially support successful implementation outcomes. CFIR as a multilevel framework identifies implementation determinants, from the individual recipients of the intervention to the implementers, the leadership, the organization and beyond. Recognizing that implementation of FICare is a complex and multidimensional phenomenon, with multiple interacting influences, will enable overcoming implementation barriers and leverage facilitators.

### 4.4. Limitations

The results shown are limited to data gathered from 55% of level II NICUs in Ontario. Although it is a reasonable response rate [24], we might have a response bias where only leadership interested in implementing FICare responded to the surveys. Hence, we cannot presume that these responses are reflective of all sites. Moreover, not all units completed both questionnaires despite reminders and requests being sent.

## 5. Conclusions

This study has confirmed the interest of many Ontario level II NICUs in implementing FICare. The survey results indicated that sites varied widely in their readiness to implement and maintain FICare. Their readiness depends on the level of NICU preparedness, including the NICU’s physical layout, environment, and space, as well as the capacity to implement FICare, the population they serve, and their geographical location. The results of this study will allow for the development of an FICare intervention and an implementation plan that overcomes the identified barriers and leverages the facilitators.

## Figures and Tables

**Table 1:** NICU characteristics identified from the Site Resource Survey.

NICU Characteristics	Frequency (%)
Duration since NICU establishment (years)	
<10	5 (26)
10–20	10 (53)
>20	4 (21)
NICU beds	
<20	14 (74)
≥20	5 (26)
Admissions in 2022	
<500	13 (68)
500–1000	5 (26)
No Response	1 (5)
Average Occupancy rate	
<50%	5 (26)
50–80%	11 (58)
>80%	1 (5)
No Response	2 (11)
Population served ^1^	
Suburban	17 (89)
Rural	12 (63)
Inner city	11 (58)
New Immigrant ^2^	26 (32)
Design of the unit	
Open Concept	11 (55)
Single family room	5 (25)
Room with 4–6 babies	1 (5)
Mixed ^3^	3 (15)
Professional Parent Support ^4^	
None	1 (5)
Yes	13 (69)
Consultation only	5 (26)
Facilities for families	
No Response	1 (5)
Yes	16 (84)
Only comfortable seating	2 (11)
Number of patient days 2022	
Average (SD)	3703 (2654)
Median (25th–75th)	2937 (2028–5282)
Average length of stay 2022	
Average (SD)	8.9 (2.6)
Median (25th–75th)	9 (7–10.8)

^1^ Units were able to identify more than one population. ^2^ New immigrant refers to someone who has immigrated to Canada within the past five years. ^3^ Mixed was defined as any unit with any combination of open concept, single-family room, or room with 4–6 babies. ^4^ Professional parent support was defined as support from a social worker, parent resource nurse, psychiatrist (perinatal), psychologist, paid parent partner, veteran parent volunteers, or chaplaincy/spiritual care.

**Table 2:** Support for implementing FICare from a leadership perspective, based on Leadership Survey.

	Agree N (%)	Neutral N (%)	Disagree N (%)
**General attitudes**			
The work climate in our unit is supportive of family centered care practices	23 (95.8)	0 (0.0)	1 (4.2)
Our staff to patient ratio is adequate to support family centered care	15 (62.5)	5 (20.8)	4 (16.7)
We provide good opportunities for staff development	20 (83.3)	4 (16.7)	0 (0.0)
Our interdisciplinary team works well together	24 (100)	0 (0.0)	0 (0.0)
Our hospital leadership will support our unit implementing practice changes to support families	23 (95.8)	1 (4.2)	0 (0.0)
Our hospital prioritizes patient engagement and in particular families of infants	20 (83.3)	3 (12.5)	1 (4.2)
Our organization regularly surveys NICU families	18 (75.0)	2 (8.3)	4 (16.7)
There are financial resources available to support new initiatives in our hospital	6 (25.0)	8 (33.3)	10 (41.7)
**Unit procedures**			
Our current NICU admission process disrupts continuous parent engagement in their baby’s care from birth	8 (33.3)	6 (25.0)	10 (41.7)
Our institutional (hospital) family presence/visitation policies are a barrier to parents being present with their baby	1 (4.2)	0 (0.0)	23 (95.8)
Our NICU specific family presence/visitation policies discourage prolonged family presence	0 (0.0)	0 (0.0)	24 (100.0)
Our current shift handover practices require parents to leave the unit	3 (12.5)	1 (4.2)	20 (83.3)
Our current structure of daily patient bedside rounds makes it difficult for parents to participate	7 (29.2)	2 (8.3)	15 (62.5)
Our current unit policies limit parent participation in their baby’s care	0 (0.0)	4 (16.7)	20 (83.3)
**Unit Environment:**			
The physical layout of our NICU provides adequate space for parents at their baby’s bedside	13 (54.2)	1 (4.2)	10 (41.7)
Our unit environment is designed to encourage parents to be present with their baby, i.e., comfortable chairs, breast pumps, etc.	14 (58.3)	6 (25.0)	4 (16.7)
Our NICU has a space for families to socialize or attend education sessions	10 (41.7)	1 (4.2)	13 (54.2)
**Project Support**			
We are interested in implementing a care model such as FICare grounded in family centered care principles	22 (91.7)	1 (4.2)	1 (4.2)
We will be able to support unit/discipline champions to facilitate the uptake of this care model	19 (79.2)	3 (12.5)	2 (8.3)
We will be able to support the staff education necessary to sustain FICare	18 (75.0)	4 (16.7)	2 (8.3)
There is capacity within our NICU to implement this care model successfully given the above considerations and follow up (staffing, policies, unit champions, ongoing training, and educational support)	18 (75.0)	2 (8.3)	4 (16.7)
There is unit and institutional interest and support for implementing and maintaining FICare	18 (75.0)	6 (25.0)	0 (0.0)
A standardized family centered care model such FICare would create cost savings for the Women and Children’s program	10 (41.7)	11 (45.8)	3 (12.5)

**Table 3:** Hospital Policies Encouraging Parents’ Participation.

Hospital Policy	Not Implemented N ^1^ (%)	Implemented N ^1^ (%)
Parents present on rounds	2 (11.1)	16 (88.9)
Skin-to-skin contact	0 (0)	18 (100)
Baby care activities	0 (0)	18 (100)
Develop Care Plans with their bedside nurse	4 (22.2)	14 (77.8)
Participate in decision making/discharge planning	0 (0)	18 (100)

^1^ A total of 18 hospitals responded to this section in the site resource survey.

**Table 4:** Characteristics of NICU Staff and Families identified by NICUs as important for consideration when implementing ON-FICare.

	Number of Units N (%)
**Characteristics of Staff ^1^**	
New graduate nurses	14 (58.3)
Part-time working nurses	7 (29.2)
Rapid Staff turnover	7 (29.2)
**Characteristics of Families ^1^**	
Families who do not speak English as their first language	17 (70.8)
Families who have cultural practices that preclude their ability to be present at the hospital	15 (62.5)
Families with food insecurity	14 (58.3)
Families with housing insecurity	16 (66.7)
Families with other children and lack of extended family or social support	22 (91.7)
Single mothers	16 (66.7)
Families with limited access to transportation	5 (20.8)
Population of substance abuse	4 (16.7)

^1^ Units were able to select more than one characteristic.

**Table 5:** Availability of Parent Volunteer Support.

	Available N ^1^ (%)	Unavailable N ^1^ (%)
Parent advisory committee	3 (16.7)	15 (83.3)
Parents on committees	8 (44.4)	10 (55.6)
Parent-to-parent support	4 (22.2)	14 (77.8)
Veteran parent (parent buddy)	5 (27.8)	13 (72.2)
Parent involved in Staff education	3 (16.7)	15 (83.2)
Paid parent	0 (0)	18 (100)

^1^ A total of 18 hospitals responded to this section in the site resource survey.

**Table 6:** Difficulties NICUs anticipate in implementing ON-FICare.

	Difficult N (%)	Neutral N (%)	Easy N (%)
Integration of Parents	5 (20.8)	5 (20.8)	14 (58.3)
Standardized Education	4 (16.7)	3 (12.5)	17 (70.8)
Coaching Parents	0 (0)	1 (4.2)	23 (95.8)
Support Parent Participation	5 (20.8)	0 (0)	19 (79.2)
Support Parent Presence	13 (54.2)	6 (25.0)	5 (20.8)

## Data Availability

The data presented in this study are available on request from the corresponding author due to privacy.

## Supplementary Materials

The following supporting information can be downloaded at: https://www.mdpi.com/article/10.3390/children12111548/s1, S1: Leadership and site resource surveys.

## Abbreviations

The following abbreviations are used in this manuscript:

NICU	Neonatal Intensive Care Unit
FICare	Family Integrated Care
ON-FICare	Ontario FICare
cRCTs	Cluster Randomized Controlled Trials
CFIR	Consolidated Framework for Implementation Research
