# Supplementary material for: Facilitators and Barriers to the Implementation of Family Integrated Care in Ontario Level II Neonatal Intensive Care Units
Source: Children (Basel). 2025 Nov 16;12(11):1548. doi: 10.3390/children12111548 (PMC12651761; doi:10.3390/children12111548)
Supplement: Supplementary file 1 [file children-12-01548-s001.zip › ON-FICare Leadership Survey.pdf]

Ontario Family Integrated Care (ON-FICare): a study of the facilitators and barriers in Level II Neonatal Intensive Care Units (Level 2 NICU's)

Leadership survey

Family Integrated Care (FICare) is an operational model developed to support the engagement of parents in the care of their infant while in the NICU as a cornerstone of family centered care. Globally, there is considerable evidence of the positive effects on infants and parents of adopting FICare in Level 3 NICUs. Adapted for Level 2 NICUs, Alberta FICare demonstrated a reduction in infant length of stay of 2.5 days without concomitant increase in emergency department visits or readmissions. With positive results from the clinical trial, Alberta FICare was scaled and spread to all 14 NICUs (level 2, level 3, and surgical) in Alberta with sustained reduction in infant length of stay and in emergency department visits and readmissions. As part of our recovery from COVID, there is currently some interest in supporting Level 2 NICU's in Ontario to implement FICare. The aim of this research study is to conduct an in-depth exploration of what might be needed to successfully implement FICare in these units, i.e. to understand your perspectives of the facilitators and barriers to implement or further support this model of care in your Level 2 NICU. The eventual goal is to use this knowledge as the basis of a future proposal to implement FICare province-wide.

Site Name: ____

Individual completing this questionnaire: ____

Individual's role: ____

Pronoun: _________________

Contact Information:

Email:____________________________________ Phone number:_____________________________

If your NICU were to be supported to adopt FICare in the next 12 months, please rate the following factors using a 5 point Likert scale "Strongly disagree" "Disagree" "Neutral" "Agree" "Strongly agree".

	Strongly Disagree	Disagree	Neutral	Agree	Strongly Agree
General attitudes					
The work climate in our unit is supportive of family centred care practices					
Our staff to patient ratio is adequate to support family centred care					

We provide good opportunities for staff development					
Our interdisciplinary team works well together					
Our hospital leadership will support our unit implementing practice changes to support families					
Our hospital prioritizes patient engagement and in particular families of neonates/infants					
Our organization regularly surveys NICU families					
There are financial resources available to support new initiatives in our hospital					
Unit procedures					
Our current NICU admission process disrupts continuous parent engagement in their baby's care from birth					
Our institutional (hospital) family presence/visitation policies are a barrier to parents being present with their baby					
Our NICU specific family presence/visitation policies discourage prolonged family presence					
Our current shift handover practices require parents to leave the unit					
Our current structure of daily patient bedside rounds makes it difficult for parents to participate					
Our current unit policies limit parent participation in their baby's care.					
Unit Environment:					
The physical layout of our NICU provides adequate space for parents at their baby's bedside					

Our unit environment is designed to encourage parents to be present with their baby i.e. comfortable chairs, breast pumps etc					
Our NICU has a space for families to socialize or attend education sessions.					
<u>Project Support</u>					
We are interested in implementing a care model such as FICare grounded in family centered care principles					
We will be able to support unit/discipline champions to facilitate the uptake of this care model.					
We will be able to support the staff education necessary to sustain FICare					
There is capacity within our NICU to implement this care model successfully given the above considerations and follow up (staffing, policies, unit champions, ongoing training, and educational support)					
There is unit and institutional interest and support for implementing and maintaining FICare.					
A standardized family centered care model such FICare would create cost savings for the Women and Children's program					

Why would your hospital be a good place to implement FICare? Select all that apply

- Support from leadership to implement FICare
- Staff motivated to integrate parents into the multidisciplinary team
- Sufficient space to accommodate parents at their infants' bedside
- Sufficient policies to facilitate parental presence
- Sufficient resources to support parents
- Medical staff support FICare
- Nursing staff support FICare
- Allied health staff support FICare
- Healthcare professionals have designated professional development time (e.g., join training sessions, complete brief online training modules)
- Staff would be willing to complete brief online FICare learning modules during 'down time' on their shift

An important part of implementing FICare is providing support to families so they can be integrated into their baby's care through parent education, coaching and engagement in medical rounds

Please rate level of difficulty you anticipate on implementing the following. Rate on 5-Likert scale "Very difficult" "Somewhat difficult" "Neutral" "Somewhat easy" "Very easy"

	Very difficult	Somewhat difficult	Neutral	Somewhat easy	Very easy
Integration of parents into the multidisciplinary care team					
Providing standardized education pathways for parents					
Providing coaching to parents to provide non-medical care for their baby as soon as they are ready and willing after admission.					
Support parent participation in daily bedside rounds					
Supporting parental presence at the infant's bedside 24/7					

Are there characteristics of families that you serve that you think need to be highlighted in the implementation of FICare at your site. Please tick below what might apply and add others that we need to know about

- families who do not speak English as their first language
- Families who have cultural practices that preclude their ability to be present at the hospital
- Families with food insecurity
- Families with housing insecurity
- Families with other children and lack of extended family or social support
- Single mothers
- Others

Are there characteristics of staff that work at your hospital that you think may need to be considered when planning to implement FICare at your site? Please indicate if any of the characteristics below may apply or we need to consider something else

- Percentage of new graduate nurses
- Part-time working nurses
- Rapid Staff turnover
- Other, ______________________

Who do you think would be the key stakeholders in your institution to be involved in the implementation steering team? List all that applies

____
____
