# Supplementary material for: Facilitators and Barriers to the Implementation of Family Integrated Care in Ontario Level II Neonatal Intensive Care Units
Source: Children (Basel). 2025 Nov 16;12(11):1548. doi: 10.3390/children12111548 (PMC12651761; doi:10.3390/children12111548)
Supplement: Supplementary file 1 [file children-12-01548-s001.zip › ON-FICare Site Resource Survey.pdf]

<u>Site Resource Survey</u>

In order to develop FICare program that suits your centre's needs please complete the below questionnaire to the best of your knowledge.

Site Name: ____

Individual completing this questionnaire: ____

Individual's role: ____

Pronoun: _______________

Contact Information:

Email:______________________________ Phone number:________________________

Site information

Years in current NICU:
Number of NICU beds: ______
Number of Funded NICU beds: ______
Number of admissions in 2022: __________________
Average bed Occupancy rates: ______
NICU annual patient days in 2022: ______
Average length of stay: ______
Patient Population demographics (Please select all that applies):

- ☐ Inner city
- ☐ Suburban
- ☐ Rural
- ☐ New immigrant

<u>Design of the unit:</u>

- o Open concept
- o Single room
- o Room with 4-6 babies
- o Other, explain: _____________

<u>Staff support:</u>

Number of Neonatologists: ______
Number of Paediatricians: ______
Number of Family doctors: ______
Number of physician assistants: ______
Number of Nurse Practitioners: ______
Nursing staff:

- o Number of Fulltime Nurses: __________
- o Number of Part-time Nurses:__________
- o Number of Registered Practical Nurses:_______

Nurse to patient ratio (describe as it may be based on acuity): ______________________

Inter-professional support

Please indicate the inter-professional support that is available in your unit:

Staff	Availability	If Availability is "Dedicated resource", enter # of FTE's
Lactation consultant	Drop down list:<br>• Dedicated resource<br>• Available for consultation<br>• Not available	
Occupational therapist		
Physiotherapist		
Developmental care specialist		
Dietician		
Respiratory therapist		
Pharmacist		
Nurse educator		
Parent resource nurse		
Paid parent partner		
Veteran Parent Volunteers		
Chaplaincy/ spiritual care		
Psychiatrist (perinatal)		
Psychologist		
Social worker		
language services/ interpreters		
Medical residents		
Medical/nursing/other students		

Access to the unit:

Parental presence policy
- Open at all times,
- Limited hours, please describe, ________________________

Siblings presence policy,
- Open at all times
- Limited hours, please describe , ________________________
- Age restriction, please describe, ________________________
- Number at bedside, please describe ________________________

Family and friends visiting policy
- Open at all times
- Limited hours, please describe , ________________________
- visiting only when parents present
- Number at bedside, please describe ________________________

Educational support for parents

Does your centre have any of the following resources available for parents?

Orientation binder or other admission information:
□ No □ Yes, if yes, please give a detailed description of the topics included in the resource:
____

Written or on-line educational material
□ No □ Yes, if yes, please give a detailed description of the topics included in the resource:
____

Are parents allowed access to their phones in the unit
□ No □ Yes

Is there computer/ tablet access for parents in the unit
□ No □ Yes

Breastfeeding classes
□ No □ Yes

CPR training
□ No □ Yes

Other in person: Parent education sessions
□ No □ Yes, if yes, please give a detailed description of the topics, frequency, the designation of the person teaching the session, number of participants in the session, and if delivered online or in person: ____

Facilities available to parents

Please indicate which of the facilities listed below are currently available in your unit to support parents.

**Facility**	**Available in NICU (Y/N)**	**Description**
Parent Lounge (If available, please indicate how many parents can be accommodated and what is available e.g. microwave, fridge)		
Comfortable seating space near Bedside		
Is a kitchen available for parents		
Breast pumps provided		
Available Breast pump space (Please specify, Pump room or bedside)		
Dedicated sleep room/hostel room (e.g. please indicate number of rooms/beds, bathroom available)		
Family/healthcare meeting room		
Care by parent rooms (parents room in with their infants)		
Is Parking cost subsidized?		

Current parent participation

In your unit, what are parents encouraged to do currently?

Activity	Y/N	Describe below
Be present for rounds?		
To provide skin-to-skin contact?		
Are parents encouraged to be involved in baby care activities?		
To develop Care Plans with their bedside nurse		
To participate in decision making / discharge planning		

Parent volunteer support

In your unit, do you have veteran parent (NICU graduate parent) participation in any form?

Parent participation	In Place (Y/N)	Being Planned (Y/N)	Approx. # of people
Parent advisory committee			
Parents on committees			
Parent-to-parent support			
Veteran parent (parent buddy)			
Parent involved in Staff education			
Paid parent			
